# Single-incision multi-port laparoscopic appendectomy: How I do it

**DOI:** 10.4103/0972-9941.72372

**Published:** 2011

**Authors:** Parveen Bhatia, Vinay Sabharwal, Sudhir Kalhan, Suviraj John, Jagpreet S. Deed, Mukund Khetan

**Affiliations:** Institute of Minimal Access, Metabolic and Bariatric Surgery, Sir Ganga Ram Hospital and Bhatia Global Hospital & Endosurgery Institute, New Delhi, India; 1 Department of Surgery, Jeewan Mala Hospital, New Delhi, India; 2 Institute of Minimal Access, Metabolic and Bariatric Surgery, Sir Ganga Ram Hospital, New Delhi, India; 3 Department of Surgery, Jeewan Mala Hospital and Institute of Minimal Access, Metabolic and Bariatric Surgery, Sir Ganga Ram Hospital, New Delhi, India

**Keywords:** Single-incision laparoscopic surgery, single access laparoscopic surgery, appendectomy, appendicectomy, single port access surgery

## Abstract

**INTRODUCTION:**

Single-incision laparoscopic surgery (SILS) appendectomy seeks to further minimise the trauma of parietal access of laparoscopic appendectomy.

**METHODS:**

We present our initial experience of 17 cases of SILS appendectomy which were completed using conventional laparoscopic instruments. We utiliesd a single-incision multi-port laparoscopic appendectomy (SIMPLA) technique.

**RESULTS:**

The operative time was 63 ± 20 min, blood loss 6.5 ± 5 mL, bowel movement (passing stool) occurred in 2.6 ± 0.6 days. Most patients were discharged on the first operative day on oral diet. The analgesic usage and pain scores were similar to multi-port laparoscopic appendectomy. No complications were noted at follow-up till 4 weeks and the surgical wound healed in all patients with an inconspicuous scar.

**CONCLUSION:**

Our initial experience with SILS appendectomy demonstrates its feasibility and supports the promise of minimising further the access of laparoscopic surgery. The clear advantage is its cosmetic benefit.

## INTRODUCTION

The performance of pauci-traumatic access surgery is an attractive clinical option. Single-incision laparoscopic surgery (SILS) denotes an effort to achieve this end.[1–4] Congregating disparate multi-port laparoscopic access to a single site has the potential to further reduce the surgical trauma. Our initial experience of SILS appendectomy reported here stems from this belief.

### Preoperative preparation

The patient is prepared for surgery as for a conventional multi-port laparoscopic appendectomy.

### Positioning of the patient and ports

The surgery is carried out under general endotracheal anaesthesia. The operative room set-up is optimised for SILS as the operative degree of freedom is limited in SILS. The operation room set-up and patient position is similar to that of a conventional laparoscopic appendectomy. The camera person stands close to and in front or behind the operating surgeon.

## SURGICAL TECHNIQUE

**Figure 1:**
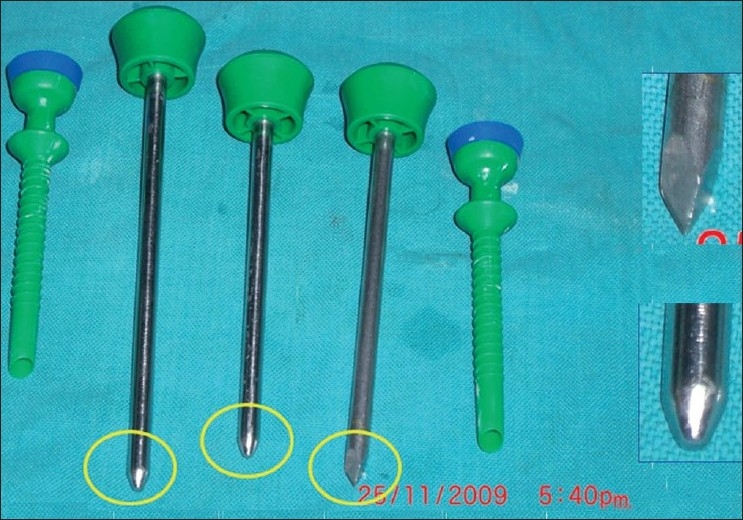
Low profile ports.

We utilise a single-incision multi-port laparoscopic appendectomy (SIMPLA) strategy and use conventional laparoscopic instruments and multiple low-profile trocars (preferably threaded trocars) [Figure 1]. A light cable that fits onto the laparoscope co-axially and has a low profile is highly desirable to allow for maximal ergonomy. Having a 10 mm and 5 mm angled scopes (30°) provides optimal vision.

### Establishing the ports

The umbilicus is infiltrated with 0.25% bupivacaine for pre-emptive anaesthesia prior to the incision. A 15 to 20-mm vertical intra-umbilical incision is made for port access. We use a Verres needle followed by a closed access method for establishing the ports. Port access can be achieved through either one of the following approaches.

**Figure 2:**
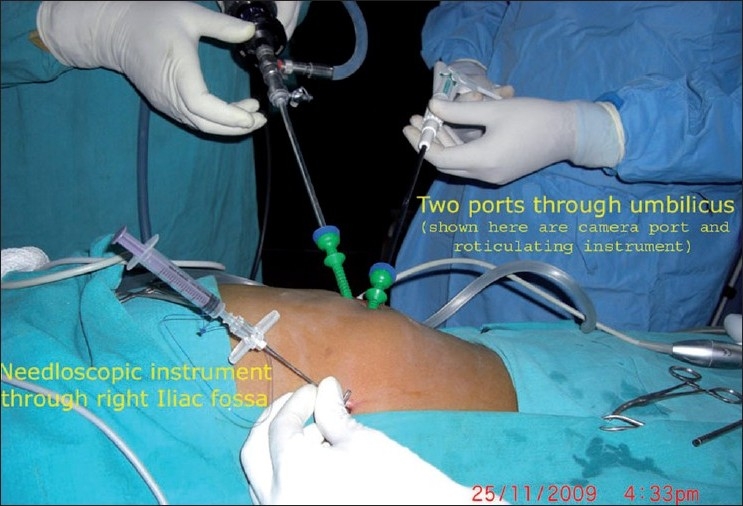
Port strategy: two ports at umbilicus and a needloscopic grasper at right iliac fossa.

**Figure 3:**
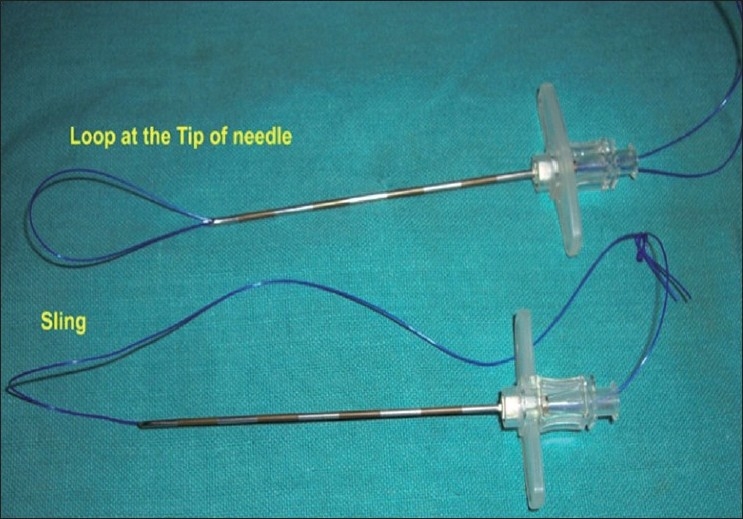
Indigenous needle-based suture-loop grasper.

**Figure 4:**
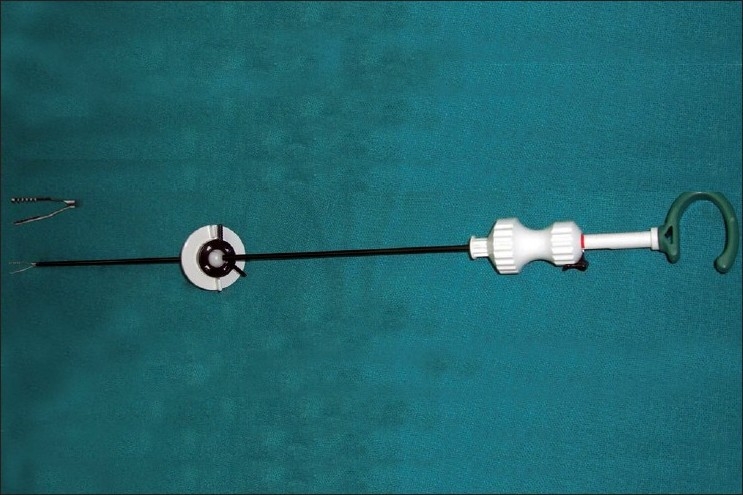
Alligator grasper.

The first approach utilises two laparoscopic ports (one 10-mm and one 5-mm port or both 5-mm ports) through the umbilical incision and one additional needloscopic instrument for assistance through the right iliac fossa or the suprapubic area [Figure 2]. The offset position of the needloscopic instrument significantly improves operating capability by replicating the instrument triangulation of conventional laparoscopy. An indigenously prepared epidural needle-based suture-loop grasper [Figure 3] or a slim alligator grasper (Stryker, Kalamazoo, MI, USA) with retractable jaws inside a 2.3 mm sheath is very useful for stabilizing the appendix [Figure 4].

The second technique utilises three laparoscopic ports placed side-by-side through the umbilical scar (one 10-mm and two 5-mm ports or three 5-mm ports).

### Localisation and exposure of the appendix

**Figure 5:**
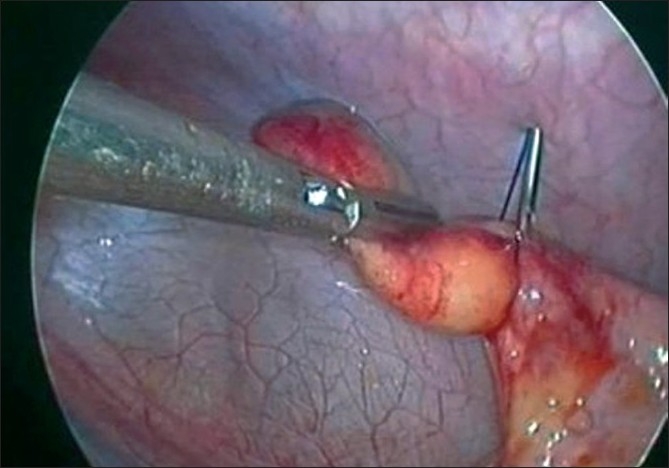
Capture of the appendix with the indigenous needle-based suture-loop grasper.

The decision to use either approach is based on the status of the appendix and surgeon comfort. Initially, a diagnostic laparoscopy is undertaken. With a Trendelenberg position and a modest right up tilt of the table the right iliac fossa is explored further. The status of the appendix is ascertained at this stage and a decision is made to either continue with SILS or to convert the procedure to a multi-port laparoscopic appendectomy. Locating the appendix can be sometimes challenging. Usually, however, the appendix can be localised utilising the same principles as employed in conventional appendectomy. The appendix is grasped with a 5-mm atraumatic grasper or with the needloscopic instrument [Figure 5].

### Control of the mesoappendix

**Figure 6:**
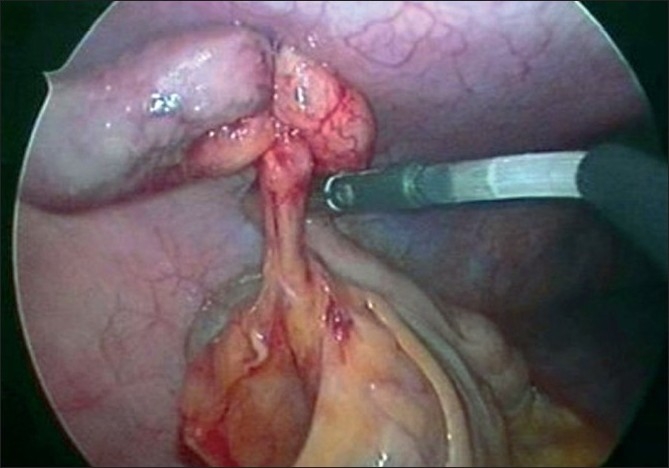
Creating a mesoappendiceal window.

**Figure 7:**
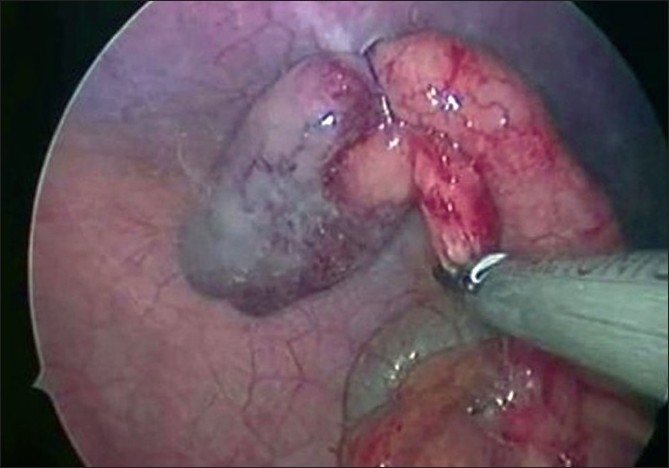
Mesoappendiceal transection with ultrasonic shears.

**Figure 8:**
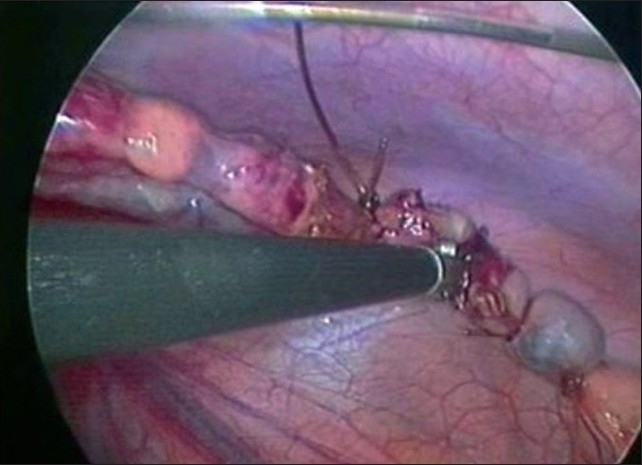
Transection of the appendiceal base

**Figure 9:**
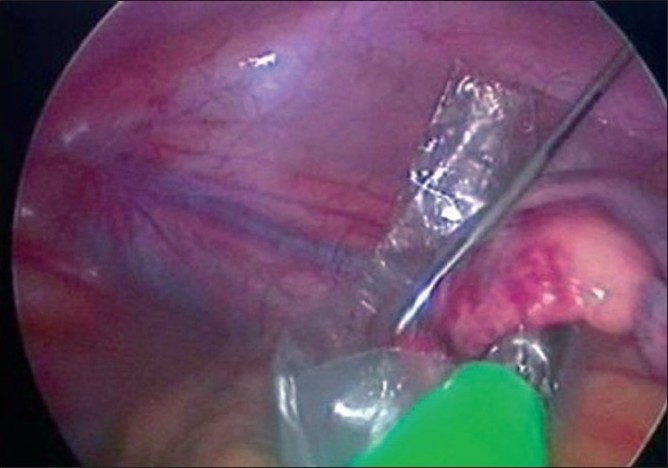
Retrieval of appendix in an Endobag

As in laparoscopic appendicectomy, care is taken to avoid avulsion of a friable/gangrenous appendix from its base. The meso-appendix is then targeted. A meso-appendicular window is created and the appendicular artery and its branches are controlled using an ultrasonic shears (Harmonic Scalpel™, Ethicon Endosurgery, Cincinnati, OH, USA) [Figures 6 and 7] or a bipolar electrocautery. The base of the appendix is then ligated thrice with catgut endo-loops and transected [Figure 8]. A 5-mm trocar can be exchanged at this stage for a 10-mm one to allow delivery of the appendix. Reinsertion of a trocar can be challenging, and needs to be done with care to avoid iatrogenic injuries. An endobag may be used for retrieval of the appendix thus avoiding port-site contamination [Figure 9].

**Figure 10:**
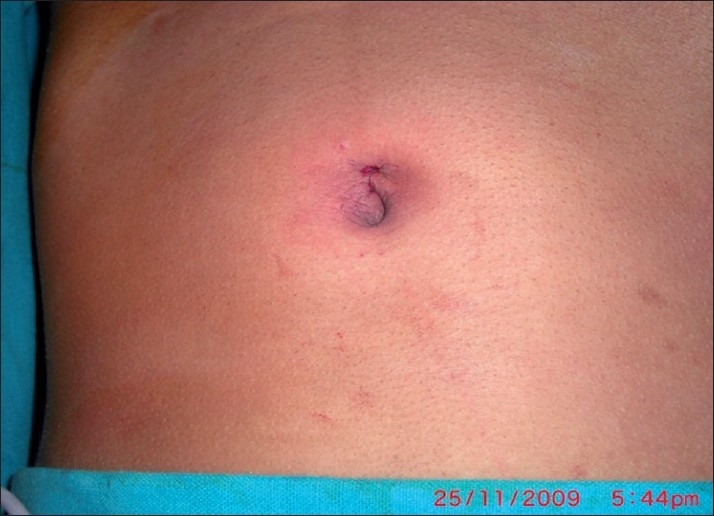
Postoperative wound.

The terminal ileum is examined by walking the bowel using atraumatic graspers. A Meckel’s diverticulum, if identified, may be similarly resected using a SILS approach. The ports are then removed under vision and the fascial incision is closed with polypropylene sutures. The wound is infiltrated again with local anaesthetic. The skin incision is closed by skin stapler or interrupted non-absorbable suture [Figure 10] and the wound dressed.

### Postoperative care

The patient is administered intravenous fluids, antibiotics, and analgesics. Oral feeds are commenced as appropriate depending on the degree of appendiceal inflammation and return of bowel function. Early mobilisation is encouraged and the patient is usually discharged on the first post-operative day.

## RESULTS

Tables 1 and 2 summarise our early experience with SILS appendectomy. Seventeen patients underwent SILS appendectomy—sixteen with acute appendicitis and one with previous appendicitis (interval procedure). The average age was 25.5 ± 12 years, and there were 12 males and 5 females. Three umbilical ports were utilized in four cases, with one of them needing an additional right iliac fossa needloscopic instrument. The remaining 13 cases were completed with two umbilical ports and an additional right iliac fossa or supra-pubic area needloscopic instrument. There were no conversions to a conventional laparoscopic approach. The mean operative time was 63 ± 20 min, blood loss was 6.5 ± 5 mL and the time taken for bowel movement (passing stool) was 2.6 ± 0.6 days. Only the patient with the perforated appendix required bilateral flank-site drains. These drained the Morrison’s pouch and pelvis and were removed on the third and fifth post-operative days, respectively. Most patients were fed and discharged on the first post-operative day. Five patients were discharged on the second post-operative day, and the case with the appendicular perforation was discharged on the fourth post-operative day. The analgesic usage and pain scores were similar to our experience with conventional laparoscopic appendectomy.

**Table 1:** Summary of our early experience

Clinical presentation	Age (years)	Gender	Ports	Additional support
Acute appendicitis	12	M	3	Indigenous grasper (at the RIF)
Acute appendicitis	16	F	3	None
Acute appendicitis	17	F	3	None
Acute appendicitis	28	M	3	None
Acute appendicitis	35	F	2	Alligator grasper (RIF)
Acute appendicitis	20	M	2	Indigenous grasper (SPA)
Acute appendicitis	24	F	2	Indigenous grasper (SPA)
Acute appendicitis	28	M	2	Indigenous grasper (SPA)
Interval appendectomy	62	M	2	Alligator grasper (RIF)
Acute appendicitis	17	M	2	Indigenous grasper (SPA)
Acute appendicitis	31	F	2	Indigenous grasper (SPA)
Appendicular perforation	22	M	2	Indigenous grasper (SPA)
Acute appendicitis	35	M	2	Indigenous grasper (SPA)
Acute appendicitis	15	M	2	Alligator grasper (RIF)
Acute appendicitis	35	M	2	Alligator grasper (RIF)
Acute appendicitis	23	M	2	Indigenous grasper (SPA)
Acute appendicitis	15	M	2	Alligator grasper (RIF)

RIF = Right iliac fossa; SPA = supra pubic area; M = male, F = female.

**Table 2:** Summary of our early experience

Mean operative time (min)	Blood loss (mL)	Bowel movement (days)	Feeding (days)	LOS (days)	Scar visibility
90	2	3	1	3	Inconspicuous
70	5	3	1	2	Inconspicuous
55	5	2	1	2	Inconspicuous
55	5	3	1	2	Mild discharge for 5 days
110	5	2	1	2	Inconspicuous
80	10	3	1	3	Inconspicuous
73	5	3	1	3	Inconspicuous
65	15	2	1	3	Inconspicuous
45	5	3	1	2	Inconspicuous
44	2	3	1	3	Inconspicuous
55	5	2	1	2	Inconspicuous
90	20	4	2	4	Drain site scars
55	5	2	1	2	Inconspicuous
50	2	2	1	2	Inconspicuous
64	5	2	1	2	Inconspicuous
40	5	2	1	2	Inconspicuous
30	5	3	1	2	Inconspicuous

LOS = Length of hospital stay.

No complications were noted on follow-up at 1 week except a transient mild serous discharge from the umbilicus in one patient. A second follow-up at 4 weeks showed good healing with an inconspicuous scar for all patients.

## DISCUSSION

Our early experience demonstrates the safety, feasibility and superior cosmetic outcome of SILS appendectomy. It, however, presents a technical and ergonomic challenge early in the learning curve. Once the curve is ascended the surgeon and the surgical team reach an intuitive level of comfort with SILS. At present, it is prudent to have a selective approach for performing SILS appendectomy in the initial stages. Our personal experience with SILS is evolving and it is clear that technology will progress much further. It must be stressed that evidence-based investigations including randomised trials will determine the proper place of SILS in future surgical practice.
